# Inclusion of edaphic predictors for enhancement of models to determine distribution of soil-transmitted helminths: the case of Zimbabwe

**DOI:** 10.1186/s13071-017-2586-6

**Published:** 2018-01-19

**Authors:** Nicholas Midzi, Blessing Kavhu, Portia Manangazira, Isaac Phiri, Susan L. Mutambu, Cremants Tshuma, Moses J. Chimbari, Shungu Munyati, Stanely M. Midzi, Lincon Charimari, Anatoria Ncube, Masceline J. Mutsaka-Makuvaza, White Soko, Emmanuel Madzima, Gibson Hlerema, Joel Mbedzi, Gibson Mhlanga, Mhosisi Masocha

**Affiliations:** 1 Department of Medical Microbiology, College of Health Sciences, University of Zimbabwe, P.O. A178, Avondale Harare, Zimbabwe; 2 Department of Geography and Environmental Science, University of Zimbabwe, P. O. Box MP 167, Mount Pleasant, Harare, Zimbabwe; 3 Ministry of Health and Child Care, P.O. Box, CY 1122 Causeway, Harare, Zimbabwe; 4 National Institute of Health Research, P.O. Box 573 Causeway, Harare, Zimbabwe; 5 University of Kwazulu Natal, Durban, 4000 South Africa; 6 Biomedical Research and Training Institute, P.O. Box CY 1753 Causeway, Harare, Zimbabwe; 7 World Health Organization, PO Box CY 348 Causeway, Harare, Zimbabwe; 8 Ministry of Primary and Secondary Education, P.O. Box CY1343, Causeway, Harare, Zimbabwe

**Keywords:** *Ascaris lumbricoides*, Hookworms, Gradient boosted model, Maxent, Species distribution, Soil-transmitted helminths

## Abstract

**Background:**

Reliable mapping of soil-transmitted helminth (STH) parasites requires rigorous statistical and machine learning algorithms capable of integrating the combined influence of several determinants to predict distributions. This study tested whether combining edaphic predictors with relevant environmental predictors improves model performance when predicting the distribution of STH, *Ascaris lumbricoides* and hookworms at a national scale in Zimbabwe.

**Methods:**

Geo-referenced parasitological data obtained from a 2010/2011 national survey indicating a confirmed presence or absence of STH among school children aged 10–15 years was used to calibrate ten species distribution models (SDMs). The performance of SDMs calibrated with a set of environmental and edaphic variables was compared to that of SDMs calibrated with environmental variables only. Model performance was evaluated using the true skill statistic and receiver operating characteristic curve.

**Results:**

Results show a significant improvement in model performance for both *A. lumbricoides* and hookworms for all ten SDMs after edaphic variables were combined with environmental variables in the modelling of the geographical distribution of the two STHs at national scale. Using the top three performing models, a consensus prediction was developed to generate the first continuous maps of the potential distribution of the two STHs in Zimbabwe.

**Conclusions:**

The findings from this study demonstrate significant model improvement if relevant edaphic variables are included in model calibration resulting in more accurate mapping of STH. The results also provide spatially-explicit information to aid targeted control of STHs in Zimbabwe and other countries with STH burden.

## Background

Soil-transmitted helminthiases are a group of neglected tropical diseases (NTDs) caused by intestinal parasites that are transmitted through faecal contaminated soil. They include *Ascaris lumbricoides*, *Trichuris trichiura*, *Necator americanus* and *Ancylostoma duodenale* [1–4]. These helminths are of a major concern to public health in tropical and sub-tropical countries where their infection is associated with devastating morbidity rates [5, 6]. About 4.5 billion are at risk of infection worldwide [7, 8] and more than 2 billion people are infected by STH [9].

The disease burden caused by these parasitic worms is enormous. In 2014, Pullan et al. [10] estimated the global numbers of people infected with hookworm, *A. lumbricoides*, and *T. trichiura*, to be 438.9 million, 819.0 million and 464.6 million, respectively. Previous estimates in 2003 by de Silva et al. [11] showed these numbers to be 740 million, 1221 million and 795 million people, respectively. In 2010, the World Health Organization (WHO) estimated that 875 million children required annual treatment with preventive chemotherapy [12]. The burden of the disease is known to be highly concentrated among the poorest socio-economic groups [12–14]. Previous estimates showed that more than 44 million pregnant women had clinical effects from hookworm-associated anaemia [15]. Hookworm-associated anaemia is known to result in the loss of 39 million disability-adjusted life years per year [16].

Based upon on the public health significance of STH, the WHO has urged member states to ensure access to essential drugs for treating STH infections in all health services in endemic areas and groups at high risk of morbidity. Such high risk groups include women and children. A goal was set to attain a minimum target of the regular administration of chemotherapy to at least 75% of all school-age children at risk of morbidity by 2010 [17]. However, to date this target has not been achieved. This is partly due to limited number of medicines and failure to precisely map the affected populations requiring treatment coupled with poor sanitation coverage and lack of a safe water supply. Global milestones for eliminating STH as a public-health problem in children were drawn by the WHO to guide efforts of member states in the fight against STH [18]. These milestones included completion of country mapping of STH by 2015. Annual mass drug administration achieving a global coverage of at least 75% by 2020 was stipulated [18]. Considering how widespread STH infection is globally, it is therefore surprising that the disease still remains neglected.

In sub-Saharan Africa, STHs have been found to be widely distributed [19–21]. However, spatially explicit information on the distribution of specific parasitic nematodes at country level remains scarce. Previous research has provided insight into the spatial epidemiology of the STHs [22, 23]. It is known that the infective stage of these nematodes is found in faecal contaminated environments especially moist and warm soils [23]. Regarding *A. lumbricoides*, fertilised eggs are known to undergo maturation in the soil for them to become infective. Hookworm eggs also hatch in the moist soil and the larvae moult twice to become infective larvae [24] that move up to the upper layers of soil to infect human hosts [7]. People typically become infected after ingesting a fully developed *A. lumbricoides* egg and/or after their skin is penetrated by third-stage hookworm larvae [25, 26]. It follows that the density of infective eggs and larvae in the soil correlates with STH exposure and risk. Thus, accurate modelling and mapping of the spatial distribution of STHs should consider edaphic variables that drive egg development for *A. lumbricoides* and are suitable for the survival of hookworm larvae.

Previous work used species distribution models (SDMs) to explore the distribution of common STH parasites in various countries including Sierra Leone [27], Kenya [28], Nigeria [22], China [29], Bolivia [30] and Brazil [31]. While most SDMs used a combination of several bioclimatic and social-economic variables as co-determinants [23, 27, 32], edaphic variables were overlooked, despite playing an important role in STH ecology and infection. There are, however, a few studies which included edaphic variables to model STHs [22, 29, 30]. In Zimbabwe, Chandiwana [33]. Described the distribution of soil-transmitted helminths (STH) using samples collected for the parasitological diagnosis of *Schistosoma mansoni*. The study reported a prevalence of 1.6% for hookworms and of 0.5% for *A. lumbricoides*. *Trichuris trichiura* was not reported [33]. The study further observed that the majority of infected children were found in the Northeast, the Zambezi Valley, the Central and Southeast low-veld areas of the country. It was, however, highlighted that the data needed to be considered with caution since the stool specimens had been collected for *S. mansoni* diagnosis and the methodology might not have been suitable for STH [33]. A recent study by Midzi et al. [20] indicated a combined prevalence of 5.5% for STH. At the species level, hookworms, *A. lumbricoides* and *T. trichiura* had the prevalence of 3.2%, 2.5% and 0.1%, respectively. The distribution of STH followed the trend as described previously [33].

Although these studies represent important progress with regard to linking the ecological theory with SDM techniques to better understand STH distribution, the studies did not report on the relative importance of edaphic variables, nor did they assess and quantify how model performance changed with the inclusion of edaphic variables. This is an important research gap that needs to be filled as a preamble to generating spatially explicit information showing in-country variability in the distribution of STHs to aid disease control and safeguard public health.

Therefore, our study tested the hypothesis that the inclusion of edaphic variables such as soil moisture in SDMs increases model performance in the spatial prediction of the distribution of STHs. Using Zimbabwe as a case study, the performance of ten SDMs comprising a set of environmental plus edaphic variables was compared to that of SDMs calibrated with environmental variables only. Since spatial prediction varies depending on the choice of variables and modeling method selected [34], it was therefore necessary to run multiple SDMs in search for the evidence for and against the above hypothesis.

## Methods

### Study area

The parasitological data used in this study were collected from primary school age children (age range 10–14 years) living in 71 districts distributed among Zimbabwe’s eight rural and two metropolitan provinces [20]. A sample of 15,818 children was calculated using EPI Info 6 statistical package (Epi Info version 6, Centers for Disease Control and Prevention, Atlanta, GA 30333) using 37% as the assumed mean prevalence of schistosomiasis and the error margin of 0.75% (see [20] for detailed information about study areas, subjects and sampling).

**Fig. 1:**
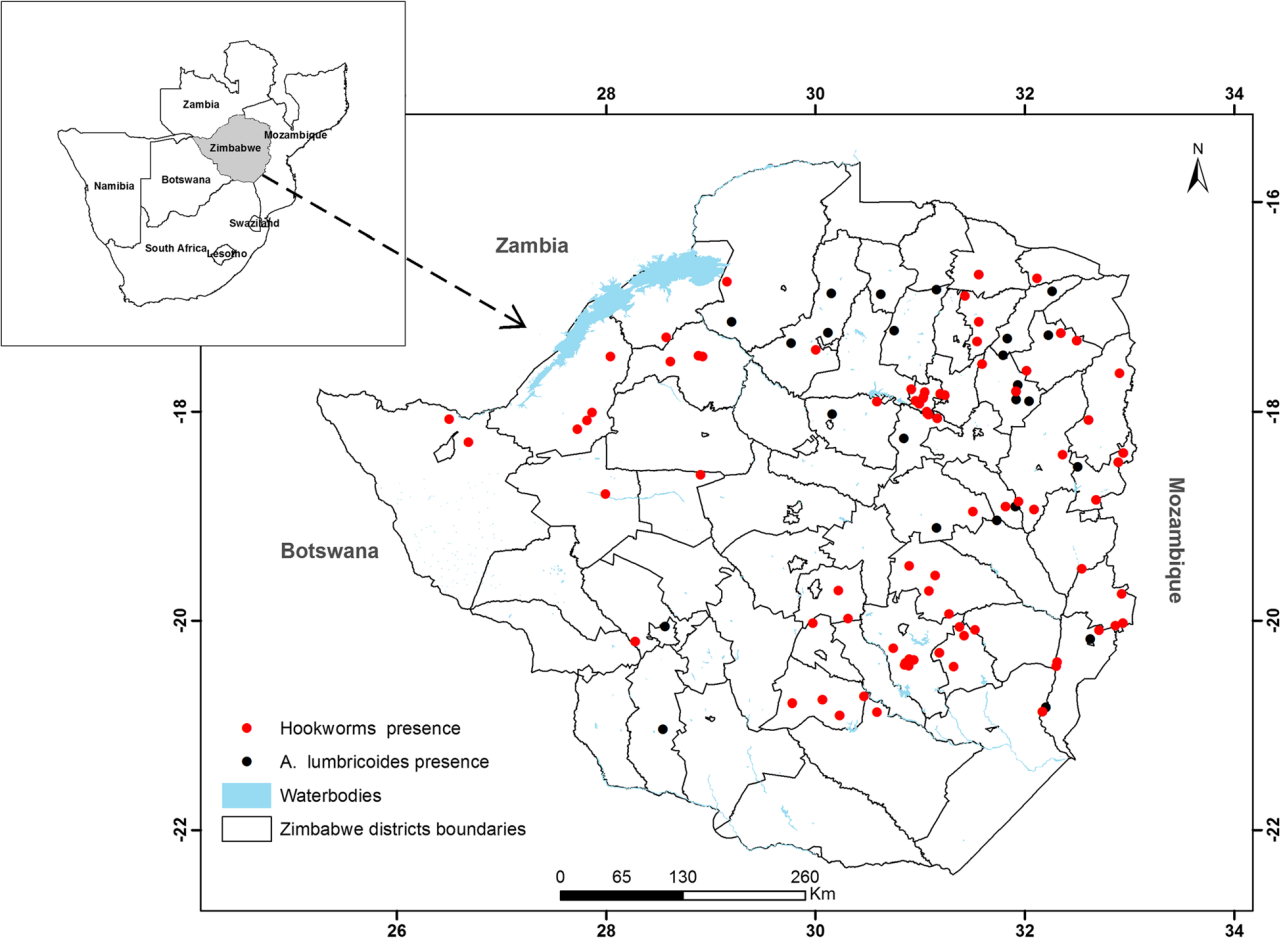
Location of Zimbabwe. *A. lumbricoides* and hookworm data are overlaid as solid circles within administrative district boundaries

To optimise health delivery, the Ministry of Health and Child Care (MOHCC) classifies 63 of the country’s 89 administrative districts as rural-based districts. The remainder are contained in the two metropolitan provinces, Harare and Bulawayo. However, it should be noted these 63 rural districts are part of the 89 districts recognised by the Government of Zimbabwe as political boundaries for enhancing local governance. Thus, the parasitological data used in this study was collected in almost all rural districts which comprise the spatial planning domain for disease surveillance and management at national scale. When writing this manuscript, the authors considered all the 89 administrative districts in order to demonstrate the important role of remote sensing and GIS technology in predicting the risk of transmission/infection with STH in which case the parasitological data could be ascribed to 71 districts where it was collected in the previous study [20]. Zimbabwe stretches from latitudes 15°37′–22°24′S and lies between longitudes 25°14′–33°04′E (Fig. 1). The country is 390,575 km^2^ in area. It borders with Zambia, Mozambique, Botswana and South Africa in the north, east, west and south, respectively. The total population was estimated at 13,061,239 in the recent census survey [35]. Altitude ranges from 300 m to 2500 m above sea level [36].

Zimbabwe has a subtropical climate, with mean monthly maximum temperature ranging from 15 °C in July to 24 °C in November. Total annual rainfall ranges from 400 mm to 1000 mm [37]. The country has assortment variety of soil types ranging from sodic and salliatic soils in the north, ferrialistic soils in the south, paraferrallistic and ortheferrilitic in the east, to regosols and Kalahari sands in the west [38, 39]. The vegetation is dominated by dry miombo woodlands in the central and east regions of the country [40]. Mopane woodlands dominate in the lowveld regions located in the northern and southern areas [41].

### STH occurrence data

Geo-referenced data for *A. lumbricoides* and hookworms collected during a national cross-sectional survey at randomly selected schools in Zimbabwe during 2010–2011 [20] were used to calibrate the SDMs. The survey targeted primary schools located in 71 of the recognised 89 administrative districts in Zimbabwe including the major urban centres of Harare, Chitungwiza and Bulawayo [20]. The prevalence of STHs was determined using the formol ether concentration and the Kato-Katz smear techniques as explained in [20]. A positive result for *A. lumbricoides* and hookworm eggs from either of the two techniques was used as an indicator for presence of these parasites among sampled school children [20].

### Environmental and socio-economic variables

A total of six environmental and demographic variables were used to model the spatial distribution of *A. lumbricoides* and hookworms in Zimbabwe. These environmental variables were: the moderate resolution imaging spectroradiameter (MODIS) monthly daytime and night-time land surface temperature (LST), annual average precipitation (AVP), MODIS normalised difference vegetation index (NDVI), human population density (HPD) and the distance from perennial water bodies (DPW). These environmental variables were selected as they have been found useful for predicting the distribution of STH [22, 29].

In brief, monthly LST daytime and night-time datasets were derived from infrared radiances measured with the MODIS aqua and terra sensors for the period January to December (both years). The datasets were accessed from the Land Processes Distributed Active Archive Centre (LP DAAC) operated by the United States Geological Survey (USGS) at https://lpdaac.usgs.gov/. Monthly LST daytime and night-time datasets were separately clipped by the polygon map of Zimbabwe, added together and divided by 12 to obtain the annual average monthly LST. AVP was calculated from gridded monthly rainfall data for the years 2010 and 2011. These rainfall data were downloaded as raster grids from the Climate Hazards Group InfraRed Precipitation with Station data (CHIRPS) archive at http://chg.geog.ucsb.edu/data/chirps/. The rainfall data were available at a 5 km spatial resolution. To capture the potential effect of vegetation on STH parasites distribution, MODIS monthly NDVI was used as a proxy for vegetation cover [42]. MODIS monthly NDVI (MOD13A3) was in the format of cloud-free imagery and was downloaded for the months January to December for 2010 and 2011 from LP DAAC at https://lpdaac.usgs.gov/. The monthly NDVI images covering the whole of Zimbabwe were averaged by year to match the temporal window at which STH parasitological data were collected in the field during the national survey.

The distance from perennial water bodies was used as a proxy for moisture availability [27]. Spatial data layers indicating the distribution and spatial extend of surface water bodies were downloaded from the Diva GIS website (diva-gis.org). These layers were projected from a geographical coordinate system (WGS 84) to a metric coordinate system (WGS 84/UTM zone 35). Then, DPW was calculated using the built in Euclidean distance function in ArcMap 9.3 [43]. The output map was projected back to a geographical coordinate system (WGS 84) to match the map projection used by other environmental variables. HPD was used to represent the potential influence of the distribution of human population (the host) on the occurrence of STH parasites [27]. The gridded human population density (version 4) for the year 2010 was downloaded from the Socioeconomic and Data Application Center (SEDAC) accessible at http://sedac.ciesin.columbia.edu/data/ [44]. The population density was mapped at a spatial resolution of 1 km.

### Edaphic variables

A suite of edaphic variables which included soil organic carbon, soil pH and soil moisture, was also used to further characterise the environmental niche of STH. The selection of these edaphic variables was based on previous literature on STH distribution as well as their relative importance to the biology of STH parasites [22, 30]. Data for organic carbon, bulk density, clay content, soil pH for the topsoil (0–30 cm) were downloaded from the ISRIC-WISE soil database as spatial layers [45]. These edaphic variables were made available at a spatial resolution of 5 km [46]. Long-term average soil moisture data with a coarse spatial resolution of 30 km were downloaded from Africa Soil Information Services website [47]. The information in Table 1 indicates the units, spatial resolution and sources of data for the environmental and edaphic variables used to predict STHs throughout Zimbabwe. Prior to modelling, all variables were re-sampled from their native resolution to a common 1 km spatial resolution using the nearest neighbour technique so that they could be overlayed. Thus, the distribution of STHs was modelled and mapped at a spatial resolution of 1 km.

**Table 1:** Characteristics of environmental variables considered important in predicting the distribution of STH in Zimbabwe

Variable	Units	Spatial resolution (km)	Data source	Accessible at
Gridded monthly CHIRPS precipitation	mm per month	~5.5	Climate Hazards Group	http://chg.geog.ucsb.edu/data/chirps/
MODIS monthly daytime land surface temperature (MOD11C3)	Kelvin	~5.5	NASA’s Land Processes Distributed Active Archive Center (LP DAAC)	https://lpdaac.usgs.gov/
MODIS monthly night-time land surface temperature (MOD11C3)	Kelvin	~5.5	NASA’s Land Processes Distributed Active Archive Center (LP DAAC)	https://lpdaac.usgs.gov/
MODIS normalized difference vegetation index (MOD13A3)	dimensionless (-1 to 1)	1	NASA’s Land Processes Distributed Active Archive Center (LP DAAC)	https://lpdaac.usgs.gov/
Gridded human population density	number of persons/km^2^	1	Socioeconomic and Data Application Centers	http://sedac.ciesin.columbia.edu/data/
Distance from perennial rivers	m	1	Calculated in a GIS	
Long-term average soil moisture	%	30	Africa Soil Information Services	africasoils.net
Soil pH	–	5	International Soil Reference Centre (ISRIC)	http://www.isric.org
Soil organic carbon (C) content topsoil (0–30 cm)	% C	5	International Soil Reference Centre (ISRIC)	http://www.isric.org

### Modelling distribution of STHs

To test for collinearity, pairwise correlations between predictor variables in raster data format were calculated in the R statistical package (Studio, 2012) using Pearson’s product moment correlation test. The folklore threshold value of *r* > 0.7 between predictor variables was used to eliminate correlated variables and to create a parsimonious model [48]. Elevation and bulk density were dropped from the modelling exercise because the latter was negatively correlated with organic carbon (*r* = −-0.80) and the former was also negatively correlated with night-time LST (*r* = -0.74).

Ten species distribution modelling techniques, namely the random forest (RF), gradient boosted model (GBM), surface range envelope (SRE), artificial neural network (ANN), generalised linear model (GLM), generalised additive models (GAM), classification tree analysis (CTA), multiple adaptive regression splines (MARS), flexible discriminant analysis (FDA) and MAXENT were used to separately predict the geographical distribution of *A. lumbricoides* and hookworms in Zimbabwe. All the models were run in the R statistical package using the *BIOMOD2* package [49]. Each model was run twice, first as a full model containing all eight predictors and secondly, as a reduced model comprising five variables without the edaphic variables.

### Model evaluation

BIOMOD2 was tuned to split presence data with 80% being used for model calibration while 20% were set aside for model validation [50]. Each SDM model was evaluated using the true skill statistic (TSS) and the area under the curve (AUC) of the receiver operating characteristic (ROC) curve. A model’s performance was considered poor if the ROC value was less than 0.6, good if ROC was within the 0.61–0.80 range and excellent if ROC value was > 0.80 [51]. ROC and TSS values were plotted against each other on a scatterplot to visualise variations in model performance under different sets of variables. Models that included and excluded edaphic variables were annotated as 1 and 2, respectively. The change in ROC and TSS model evaluation scores following the inclusion of edaphic predictors was separately calculated as a percentage for all the ten SDMs.

The TSS and ROC values for the ten modelling techniques were tested for normality using the Shapiro Wilk’s test. TSS and ROC scores for *A. lumbricoides* followed a normal distribution whilst those for hookworms did not follow a normal distribution. Therefore, to test for significant differences in model performances under different variable sets, an independent *t*-test was used for *A. lumbricoides,* whereas the Mann-Whitney U-test was used for hookworm data. The TSS and ROC were the response variables and model type was the categorical explanatory variable. Category (1) models comprised of model evaluation scores obtained using a set of variables which included the edaphic predictors. Category (2) comprised of model evaluation scores obtained from a variable set that excluded edaphic predictors.

### Consensus modelling of STH

Models with TSS and ROC greater than 0.5 and 0.7 respectively, were identified and used to build a consensus model for predicting the continuous distribution of *A. lumbricoides* and hookworms throughout Zimbabwe. Specifically, for each species, a consensus model was created by combining the predictions of the top three performing models with ROC > 0.7 and TSS > 0.5. The spatial predictions of the consensus distribution model were exported to geographical information system software (Arc Map 9.3) to display the distribution throughout Zimbabwe as a continuous map. The continuous probability of presence map was classified into five distinct thematic classes based on the natural breaks in the data to enhance visual contrast. To zoom in on potential presence, a threshold value of TSS ≥ 0.5 was used to generate a binary map showing potential presence of *A. lumbricoides* and hookworms for ease of communication and to aid the management of STH in Zimbabwe.

### Assessing variable importance

BIOMOD2 was calibrated to automatically compute variable importance. Variable importance was assessed only for the top three performing models. The goal was to check whether the inclusion of edaphic variables was as hypothesised. A variable was considered to be important when its value was > 0.10.

## Results

### Prevalence of STH in Zimbabwe

Results used in preparing this manuscript were obtained from the national survey conducted by Midzi et al. [20]. Of the estimated sample size (*n* = 15,818) for the national survey, 12,252 (77.5%) participants were screened for infection with any of the soil-transmitted helminthes (hookworms, *Trichuris trichiura* and *Ascaris lumbricoides*). Results from the study by Midzi et al. [20] showed the overall combined prevalence of STH of 5.5%, ranging between 0 and 18.3% in provinces, 0–45% in districts and 0–78.7% in schools. There was no significant difference in the prevalence of STH between males (7.5%) and females (6.9%) (Fisher’s exact test, *P* = 0.231). The prevalence of STH was highest in Binga district (45.5%, 95% CI: 38.46–52.67%) followed by Mutoko (43.5%, 95% CI: 35.55–51.72%) and Murehwa district (40.6%, 95% CI: 34.07–47.46%). Overall, STHs were predominantly distributed in the northern, northeastern and eastern regions and scantly distributed in the western region of Zimbabwe [20].

### Performance of SDMs for predicting STHs distribution in Zimbabwe

#### Data based on modelling of edaphic variables

**Fig. 2:**
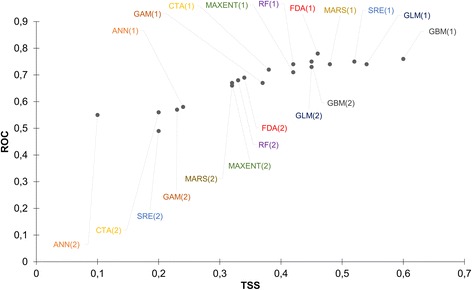
Scatterplot of TSS and ROC illustrating the performance of ten modelling techniques used to predict the distribution of *A. lumbricoides* in Zimbabwe

**Fig. 3:**
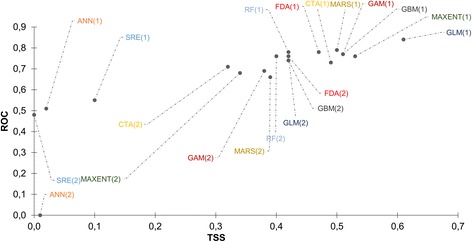
Scatterplot of TSS and ROC illustrating the performance of ten modelling techniques used to predict hookworms distribution in Zimbabwe

Model performance varied among the ten modelling techniques as illustrated in Figs. 2 and 3. Models which included edaphic variables performed better in predicting the distribution of *A. lumbricoides* compared to models that excluded edaphic variables (Fig. 2). The same pattern was observed for hookworms as illustrated in Fig. 3. Specifically, the results reveal that for both *A. lumbricoides* and hookworms, models that contained environmental plus edaphic variables yielded superior results (TSS > 0.5 and ROC > 0.75) compared to those which had environmental predictors only.

Figure 2 also illustrates that GBM, GLM, SRE outperformed other modelling techniques in predicting the distribution of *A. lumbricoides* with TSS and ROC values greater than 0.50 and 0.75, respectively. By contrast, ANN, GAM and CTA performed poorly. For hookworms, the GLM, MAXENT and GBM were the best performing models. The ANN, SRE and RF performed poorly (Fig. 3). Thus for both *A. lumbricoides* and hookworms, the GLM and GBM consistently performed well whereas the ANN performed poorly for both species with TSS < 0.3.

For *A. lumbricoides,* the results of the *t-*test confirmed significant differences in model performance between the two sets of models, i.e. the models trained with environmental variables only versus those trained with environmental plus edaphic variables (TSS: *t*_(18)_ = 3.1, *P* = 0.006 and for ROC: *t*_(18)_ = 2.48, *P* = 0.023). Similarly, hookworms results for the Mann-Whitney U-test indicated significant differences in model performance between these two sets of SDMs (TSS: *U* = 17.5, *P* = 0.01 and for ROC: *U* = 21.5, *P* = 0.029).

#### Changes in model performance

The percentage change in model performance varied among the ten modelling techniques as summarised in Table 2. The largest improvement in model performance was obtained for SRE following the inclusion of edaphic variables with a percentage increase of 160 and 53% for TSS and ROC evaluation techniques, respectively. By contrast, the lowest percentage change in model performance was obtained for GLM and GBM with the former recording a 20% change when evaluated using TSS whilst the latter recorded 1.3% change using the ROC evaluation technique.

**Table 2:** Percentage change in model performance among ten modelling techniques used to predict *A. lumbricoides* distribution in Zimbabwe

Model	TSS (2)^a^	TSS (1)^a^	% change	ROC (2)^a^	ROC (1)^a^	% change
ANN	0.10	0.24	140	0.55	0.58	5
CTA	0.20	0.38	90	0.56	0.72	29
FDA	0.34	0.46	35	0.69	0.78	13
GAM	0.23	0.37	12	0.57	0.67	18
GBM	0.45	0.60	33	0.75	0.76	1
GLM	0.45	0.54	20	0.73	0.74	1
MARS	0.32	0.48	50	0.66	0.74	12
MAXENT	0.32	0.42	31	0.67	0.71	6
RF	0.33	0.42	27	0.68	0.74	9
SRE	0.20	0.52	160	0.49	0.75	53

^a^Evaluation scores for models with environmental variables only are denoted TSS (2) and ROC (2) and those derived from a set of environmental variables plus edaphic variables are denoted TSS (1) and ROC (1)

Results in Table 3 also show that percentage change in model performance varied amongst the ten SDMs used to model the distribution of hookworms. The SRE recorded the largest percentage increase in model performance (9900%) following the inclusion of edaphic predictors when evaluated using the TSS. The ANN was also characterised by the largest increase in model performance (5000%) when evaluated using the ROC. The lowest percentage change in model performance was recorded for RF with values of 5% and 2.6% for TSS and ROC, respectively.

**Table 3:** Percentage change in model performance among ten modelling techniques used to predict hookworms distribution in Zimbabwe

Model	TSS(2)^a^	TSS (1)^a^	% change	ROC (2)^a^	ROC (1)^a^	% change
ANN	0.00	0.02	1900	0.01	0.51	5000
CTA	0.32	0.49	53	0.71	0.73	3
FDA	0.42	0.47	12	0.76	0.78	3
GAM	0.38	0.51	34	0.69	0.77	12
GBM	0.42	0.51	21	0.74	0.77	4
GLM	0.42	0.61	45	0.76	0.84	11
MARS	0.39	0.50	28	0.66	0.79	20
MAXENT	0.34	0.53	6	0.68	0.76	12
RF	0.40	0.42	5	0.76	0.78	3
SRE	0.00	0.10	9900	0.48	0.55	15

^a^Evaluation scores for models with environmental variables only are denoted TSS (2) and ROC (2) and those derived from a set of environmental variables plus edaphic variables are denoted TSS (1) and ROC (1)

#### Predicted geographical distribution of STHs in Zimbabwe

**Fig. 4:**
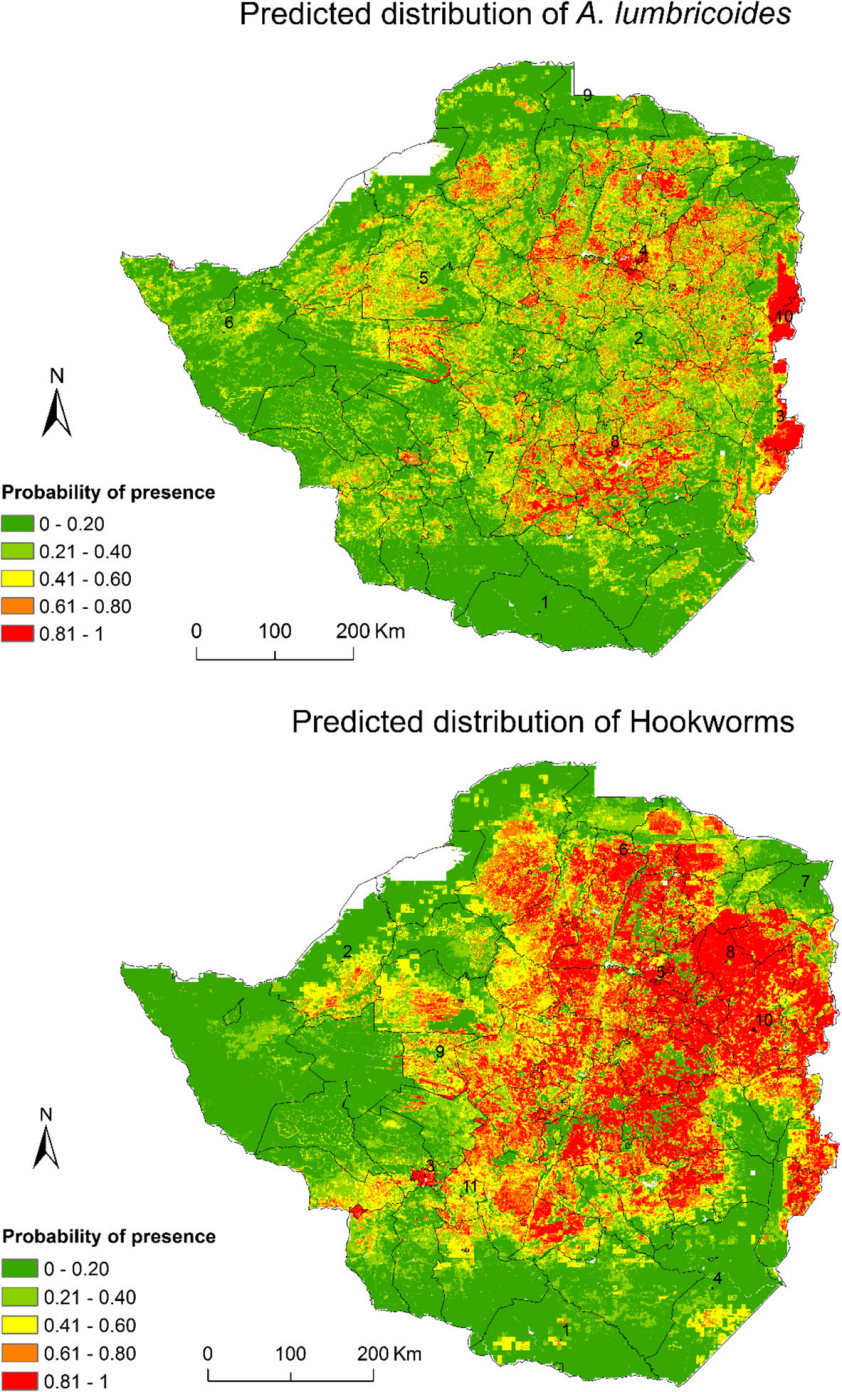
The predicted spatial distribution of *A. lumbricoides* and hookworms across Zimbabwe

The predicted probability of the presence of *A. lumbricoides* varied among the 89 administrative districts of Zimbabwe. The districts characterised by the highest probability of presence were located in the eastern parts of the country with a probability > 0.8. These included Chimanimani (3), and Mutasa (10) shown in Fig. 4. The districts located in the western, southern and the central watershed regions such as Harare (4), Gokwe South (5), Insiza (7), Masvingo (8) and Chikomba (2) were characterised by moderately high probabilities of presence. In contrast, districts at the southern, western, and northern extents of the country which included Beitbridge (1), Hwange (6) and Mbire (9) were characterised by low predicted probabilities of the presence for *A. lumbricoides*.

Similar to *A. lumbricoides,* the predicted distribution pattern for hookworms indicated the highest probabilities of presence for districts in the eastern areas of the country. The districts characterised by highest probabilities included Rusape (10), Murehwa (8), Chitungwiza (5), Guruve (6) and Bulawayo (3) as illustrated in Fig. 4. The districts situated in the western parts of the country including Binga (2), Nkayi (9) and Umzingwane (11) were characterised by moderately high probabilities of presence. Low probabilities of presence for hookworms were predicted for districts located in the eastern and southern regions of the country such as Mudzi (7), Chiredzi (4) and Beitbridge (1).

#### Spatial pattern of STHs occurrence in Zimbabwe

**Fig. 5:**
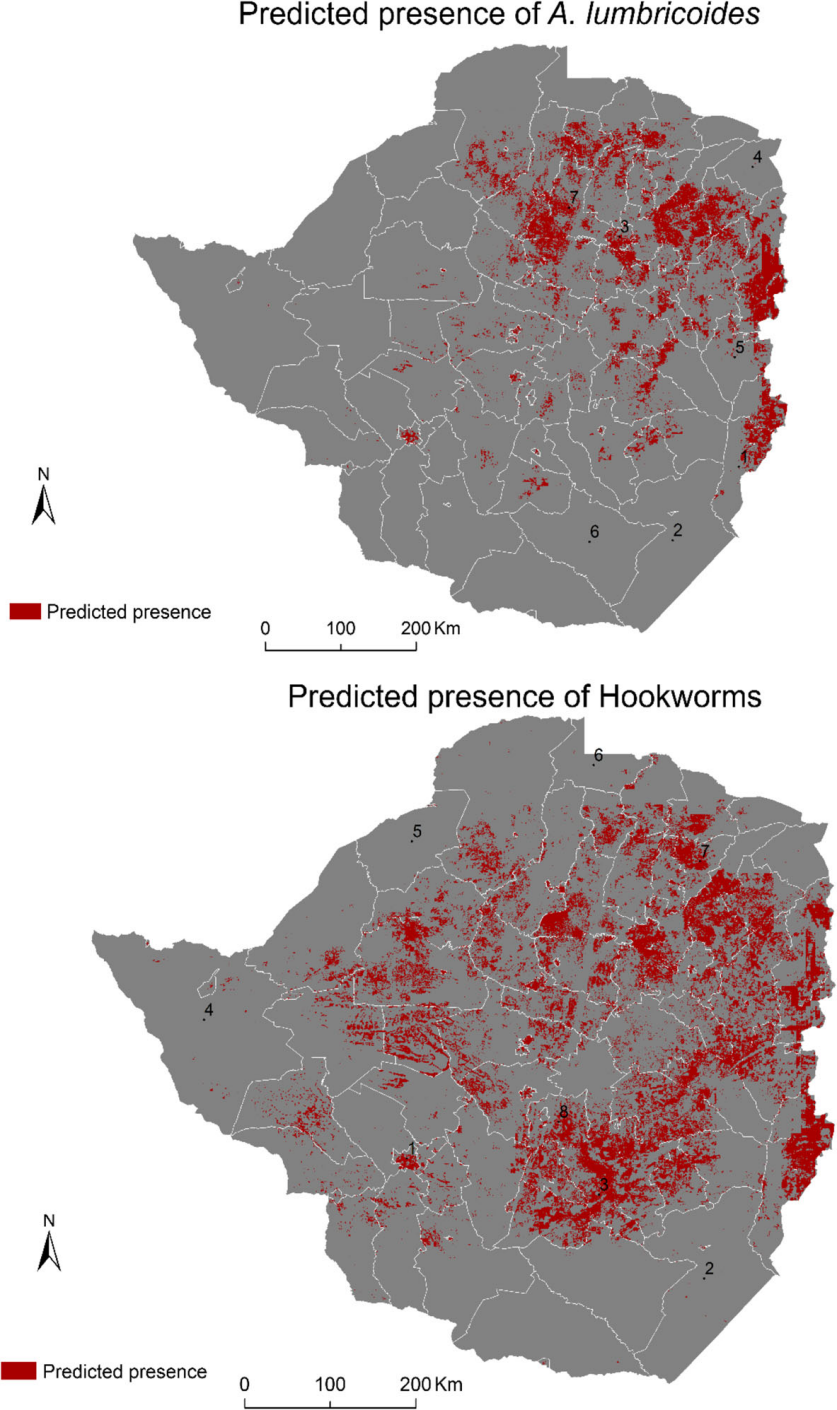
The predicted presence of *A. lumbricoides* and hookworms within and across the administrative districts of Zimbabwe

*Ascaris lumbricoides* was predicted to be occurring in 66 districts stretching from the northern to the eastern parts of the country (Fig. 5). Districts characterised by high *A. lumbricoides* presence included Chipinge (1), Zvimba (7) and Harare (3). The predicted presence for hookworms was more widespread in the country compared to *A. lumbricoides* with the species predicted as occurring in 74 districts, predominantly districts in the northern, eastern and southern parts of the country, particularly Shamva (7), Shurugwi (8), Chivi (3) and Bulawayo (1).

#### Variable importance

The results in Table 4 reveal that NDVI was consistently identified as the most important predictor for all the three top performing models. Soil pH was also an important variable for GLM and GBM followed by HPD selected as important by GBM and SRE. It was observed that for each of the top three performing models, at least one of the three edaphic variables was considered an important predictor for modelling the distribution of *A. lumbricoides*.

**Table 4:** Variables identified as important for modelling the geographical distribution of *A. lumbricoides* and hookworms in Zimbabwe

Variable	*A. lumbricoides*			Hookworms		
	GLM	GBM	SRE	GLM	GBM	MAXENT
DPW	0.147*	0.062	0.071	0.153*	0.075	0.000
Soil moisture	0.078	0.069	0.183*	0.062	0.025	0.001
Soil pH	0.551*	0.144*	0.076	0.062	0.004	0.556*
Soil organic content	0.089	0.026	0.044	0.159*	0.211*	0.131*
HPD	0.030	0.266*	0.206*	0.048	0.365*	0.261*
AVP	0.111*	0.034	0.152*	0.000	0.037	0.081
NDVI	0.379*	0.132*	0.190*	0.460*	0.233*	0.000
LST(day)	0.193*	0.079	0.161*	0.295*	0.088	0.148^*^

*Abbreviations:* *GLM* generalised linear model, *GBM* gradient boosted model, *SRE* surface range envelope, *Maxent* maximum entropy, *DPW* distance from perennial water body, *HPD* human population density, *AVP* average annual precipitation, *LST* land surface temperature

*Important predictors

With regard to hookworms, soil organic matter was identified as the most important variable for predicting hookworms by GLM, GBM and MAXENT. Similar to the results for *A. lumbricoides*, at least one of the three edaphic variables was considered important for modelling the distribution of hookworms in Zimbabwe. HPD was selected twice as an important variable for both STHs modelled. Thermal variables, in particular LST (day) and LST (night), also appeared to be influential in predicting both *A. lumbricoides* and hookworms. NDVI was also a key variable for predicting hookworms when using GLM and GBM.

## Discussion

The results of this study provide empirical support to the hypothesis that the inclusion of edaphic variables improves model performance when predicting the distribution of STHs at country scale. A consistent improvement in model performance was achieved among a wide variety of modelling techniques when edaphic variables such as organic matter content were combined with other environmental variables to make spatial predictions of *A. lumbricoides* and hookworms presence in Zimbabwe. Furthermore, the observed statistically significant percentage increases in model performance demonstrate that inclusion of edaphic predictors enhances models to determine the distribution of soil-transmitted helminths. While the inclusion of edaphic variables in modelling STH occurrences has been undertaken in China [29], Bolivia [30] and Nigeria [22], this study is the first (to our knowledge) to report superior results when comparing models calibrated using environmental plus edaphic variables to those that exclude the latter. Thus, studies that exclude edaphic variables could be either under- or over-estimating the distribution of STHs [27, 28, 32]. Although model performance consistently improved following the inclusion of edaphic predictors on all the ten SDMs and for both STH parasites, the level of improvement varied with each modelling technique. This result confirms the widely observed discrepancy among different modelling techniques and justifies the need to run several SDMs to better characterise the niche space of a target species.

In this study DPW, soil moisture, soil pH, HPD, AVP, NDVI, daytime LST and night-time LST were found to be important variables for predicting the distribution of *A. lumbricoides.* This result corroborates a previous study which documented the important role that moist and warm conditions play in promoting quick embryonation of *A. lumbricoides* [24]*.* The high importance attached to NDVI in this study suggests that the occurrence of *A. lumbricoides* is also influenced by vegetation cover. This may not be surprising as previous research reported that eggs of *A. lumbricoides* die when exposed to direct sunlight [52]. The observation that at least one of the edaphic variables proved to be important for each of the top performing models implies that edaphic variables are critical when modelling the distribution of *A. lumbricoides*. Similarly, previous studies [22, 27, 30] noted that soil pH, HPD, AVP, LST (day) and LST (night) were relatively important in predicting the distribution of *A. lumbricoides* after factoring in collinearity among predictor variables.

The observed consistency of high importance values for soil organic matter in all the top performing models are in line with the ecology of hookworms as the parasites feed on organic matter [53–55]. Thus, leaving out this edaphic variable in modelling the distribution of hookworms, likely leads to under-representation of the environmental niche within which these parasites thrive. Considering that with the advances in GIS and remote sensing technology, spatial data layers of organic matter content and other edaphic variables are now available in the public domain to modellers, the findings of this study open up opportunities to increase the accuracy of STH mapping at country scale. It is also important to note that DPW, HPD, NDVI, LST day and LST night were identified as important variables. This is in concurrence with previous studies which reported their importance in predicting the distribution of hookworms in different regions of the world [29, 30]. What makes this study different from others is the emphasis on edaphic variables, particularly soil pH and soil organic content, when predicting the distribution of hookworms in different geographical regions of the world.

From a disease management perspective, results of our study indicate a wide geographical distribution of *A. lumbricoides* and hookworms in Zimbabwe. High probabilities of presence values for *A. lumbricoides* were found in the northern and eastern districts in the country characterised by warm and moist conditions for the greater part of the year, which give rise to high vegetation cover if other factors, such as anthropogenic disturbance that change land cover, remain constant. In the case of hookworms, a wider distribution compared to that of A. *lumbricoides* was presented with highest probabilities of presence being reported in the northern, eastern and central districts of the country. Low probabilities of *A. lumbricoides* presence were found for districts in the southernmost, westernmost and northernmost districts. Since the parasitological results from Midzi et al. [20] were used in our study, it is not surprising that the findings in we observed some similarities in the distribution trend with the previous observations made at a national scale, i.e. that STH were predominantly distributed in the northern, northeastern and eastern regions, and scantly distributed in the western and south-western regions of Zimbabwe [20]. The parasitological data used by this study were from primary school children aged 10–15 years [20].

Overall, this work underlines the importance of modelling for policy decisions as this can assist in risk assessment at low cost whilst producing quick results. Specifically, geospatial technology used in this study facilitated the production of the first continuous distribution maps for two problematic STHs in Zimbabwe. These continuous distribution maps have an advantage of showing variations within and across districts in the distribution of STH parasites including some of the districts which were not sampled during the 2010/11 national survey namely Gweru, Kwekwe, Chegutu, Shurugwi, Sanyati and Mhondoro-Ngezi. Thus, the current results complement previous work in which STH prevalence was mapped using point data [20]. The results also show that the districts of Chimanimani, Nyanga, Mhondoro-Ngezi, Epworth, and Chitungwiza need to be added to the list associated with high *A. lumbricoides* prevalence. Likewise, in the case of hookworms, seven districts including Rusape, Hwedza, Nyanga, Chegutu, Mberengwa and a metropolitan province, Bulawayo, could be considered as high prevalence areas.

Although the findings from our study appear stable considering that ten modelling techniques were employed and model evaluation was based on two metrics, a limitation of the study is that other common STH species which are prevalent in Zimbabwe were not considered due to a lack of geo-referenced occurrence data. Thus, as these spatial data become available, it would be worthwhile to also test the effect of including edaphic variables on model performance when predicting the distribution of other STH such as *Trichuris trichiura*. This study was also conducted at a national scale with the aim to bolster policy formulation and hence fine scale variations in the distribution of STHs could have been missed. For instance, only distance from permanent water bodies was used to characterise the aquatic habitat of STHs but at the local scale, there are areas that get wet during parts of the year and depending on soil type and livelihoods activities (such as vegetable gardening) can provide suitable conditions for hookworms, especially in areas with poor sanitary conditions.

Another limitation of this study is that whilst the comparison of population densities in urban areas vs rural areas would act as a proxy of for the other related variables including sanitation and access to clean water, in this study we did not choose to analyse for these aspects for the following reasons: (i) a better analysis could have been accomplished if the data on these variables had been collected at the time of the study, and (ii) in Zimbabwe there are several development partners undertaking health development projects in some districts including water and sanitation provision. It is, however, unknown how these facilities are used by the communities of diverse cultures.

## Conclusions

This study has shown that inclusion of edaphic predictors enhances model performance when predicting the geographical distribution of STHs. In addition, the study produced the first continuous distribution maps for two widely occurring STHs in Zimbabwe thus, confirming their wider distribution than previously thought.
